# The influence of ageism on stereotypical attitudes among allied health students in Japan: a group comparison design

**DOI:** 10.1186/s12909-020-02439-0

**Published:** 2021-01-07

**Authors:** Yuko Fukase, Naoto Kamide, Norio Murayama, Akie Kawamura, Kanako Ichikura, Yoshitaka Shiba, Hirokuni Tagaya

**Affiliations:** 1 Kitasato University School of Allied Health Sciences, 1-15-1, Kitazato, Minami-ku, Sagamihara, Kanagawa 252-0373 Japan; 2 Faculty of Health and Sports Science, Juntendo University, 1-1 Hiraka-gakuendai, Inzai, Chiba, 270-1695 Japan

**Keywords:** Ageism in health care, Allied health students, Stereotype, Education about ageism, Random allocation

## Abstract

**Background:**

Ageism is a serious problem in medical care. The importance of ageism-related education for students has been emphasized. To determine the most effective approach to ageism-related education for allied health students, this study examined ageism among this group of students, with the hypothesis that ageism was expressed not only toward elderly adults but also toward individuals other than elderly adults.

**Methods:**

A questionnaire survey was conducted among 154 allied health students in Japan. The questionnaire involved tree drawings to evaluate the drawer’s personality and a measurement of the participants’ ageism. There were two display conditions for tree drawing. In the elderly display condition, participants were informed that the drawer was an elderly person, and in a control condition, participants were not informed of the drawer’s age. Participants were randomly assigned to each condition and were required to evaluate the drawer’s personality based on 5 personality traits. After the evaluation, all participants were required to complete the Japanese short version of the Fraboni Scale of Ageism (FSA-J).

**Results:**

The participants were 123 allied health students, 61 of whom were in the elderly display condition and 62 of whom were in the control condition. Based on the mean score on the FSA-J (*M* = 29.80), we divided the participants into a low-FSA-J group (*N* = 64) and a high-FSA-J group (*N* = 59). There was no significant difference between the display conditions on the FSA-J score. In the high-FSA-J groups, the control condition evaluated the drawer’s personality as more timid than did the elderly display condition (*F* = 4.26, df = 1, 119). For negligence, the high-FSA-J group evaluated the drawer’s personality as more negligent than did the low-FSA-J group (*F* = 4.08). For broad interests, the main effects of condition and groups were significant (*F* = 4.23).

**Conclusions:**

The results suggested that ageism indicated a negative evaluation not only of elderly adults but also of individuals other than elderly adults, and students with negative ageism might evaluate the elderly drawer more positively. We have discussed the possibility that negative ageism among allied health students in Japan might underlie these positive stereotypes.

## Background

Ageism, or a negative prejudice toward elderly adults, is a serious problem in medical care. It has been reported that a negative attitude among healthcare professionals toward elderly patients leads to low-quality care and therapy [1, 2] and elder abuse and neglect [3, 4]. In Japan, 50.2% of all patients are older [5], and ageism in health care is an urgent issue [6].

To reduce ageism, the importance of education for allied health students has been emphasized [7–12]. Ageism among young people is thought to be caused by a lack of knowledge, a lack of communication with elderly adults, aging anxiety and a fear of death [13–16]. North et al. [17] suggested that ageism among the young toward elderly adults is based on their envy of the resources, societal position, unequal sharing of government money and public space afforded to elderly adults, as well as the limited participation of elderly adults in activities usually reserved for younger people. It was reported that education based on information about elderly adults and descriptions of an intergeneration between elderly and young individuals were effective ways to reduce ageism among undergraduate students [18]. These studies argued that an effective educational program can contribute to a negative attitude toward elderly adults among allied health students.

For effective education on ageism for allied health students, it might be necessary to consider negative attitudes toward not only elderly adults but also those other than elderly adults because ageism might be associated with personality traits; for example, ageism is negatively associated with agreeableness and conscientiousness and positively associated with neuroticism [19].

Whether ageism is caused by a lack of knowledge, a lack of communication, or an unconscious fear of aging and death, ageism is nonetheless prejudice toward others. When ageism influences behavior not only toward elderly adults but also toward those other than elderly adults, education on only attitudes toward elderly adults is insufficient to reduce ageism. Therefore, this study examined ageism among allied health students, including negative attitudes toward elderly adults and toward those other than elderly adults. If a negative attitude based on ageism influences only elderly adults, then allied health students with high levels of ageism evaluated only elderly adults negatively.

## Methods

The study was conducted in accordance with the CONSORT guidelines [20].

### Design and procedure

We conducted a questionnaire survey for allied health students. Participants were required to evaluate a drawer’s personality using the tree drawing and answer the measure of participant levels of ageism.

A tree drawing is a projective test [21]. Usually, it is used to assess the drawer’s personality; however, this study used it to assess the assessor’s attitude toward elderly adults according to the suggestion that the evaluation somewhat reflected the assessor’s values [22]. There were two display conditions of the tree drawing, however, the same tree drawing was used for the two display conditions: an elderly display condition and a control condition. In the elderly display condition, participants were informed that the drawer was an elderly person, and in a control condition, participants were not informed of the drawer’s age. The questionnaire states the following in the elderly display condition: ‘This is a tree drawn by someone 65 years of age or over. Please surmise the personality of the person who drew this tree.’ The questionnaire states the following in the control condition: ‘Please surmise the personality of the person who drew this tree.’ Then, the participants were required to evaluate the drawer’s personality based on 5 personality trait terms. Both types of questionnaires were simple and randomly distributed.

After the evaluation of the drawer’s personality, all participants were required to complete the Japanese short version of the Fraboni Scale of Ageism.

### Material

#### Fraboni scale of ageism

A Japanese short version of the Fraboni Scale of Ageism (FSA-J) was used to evaluate the participants’ ageism [23]. This scale comprised 14 items measured on a five-point scale ranging from 1 (‘I don’t think so’) to 5 (‘I think so’) (range = 14–70). Higher scores indicate higher negative levels of ageism. The validity was confirmed by exploratory and confirmatory factor analysis, and the reliability was confirmed by Cronbach’s alpha [23]. The scores among allied health students tended to be lower than those among adults: the mean score and SD among nursing students in Japan was 29.1 ± 7.2 in the first year and 26.4 ± 6.0 in the fourth year [24], and the score among Japanese young men was 31.82 ± 7.78 [23].

#### A tree drawing

**Fig. 1:**
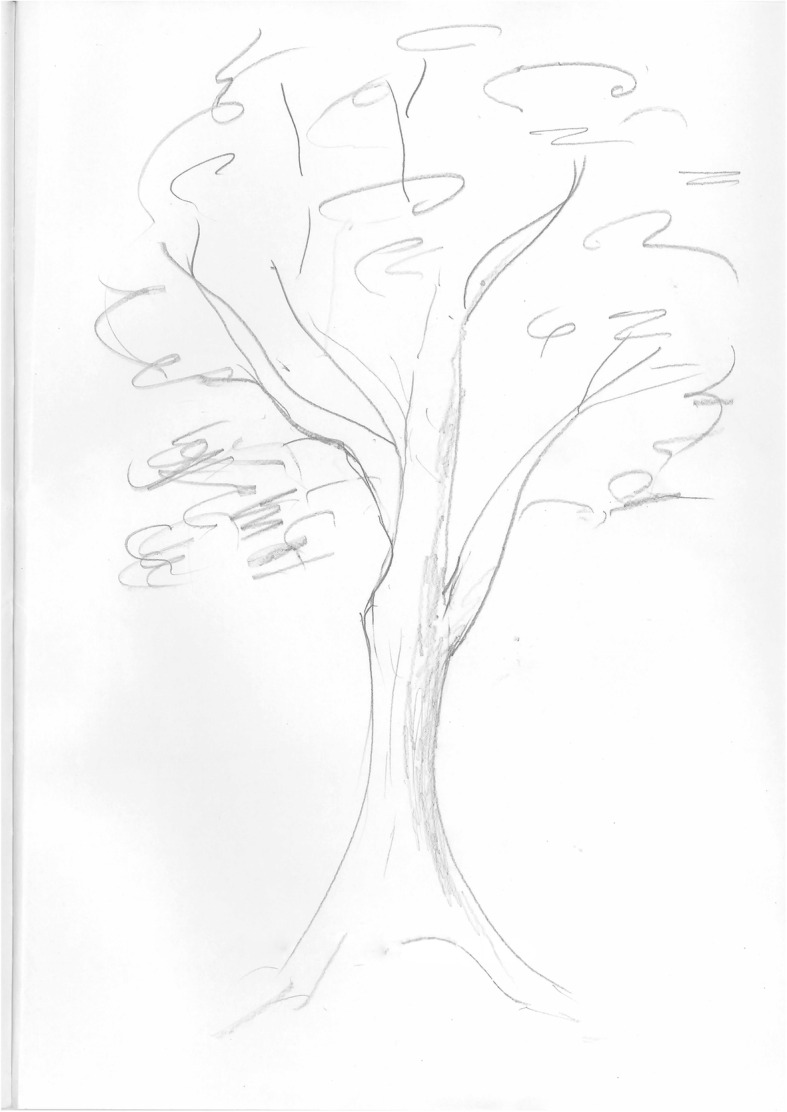
Tree-drawing used for this study

In this study, to investigate the assessor’s attitude [22], an assessor evaluates a drawer’s personality through a tree drawing based on the overall impression. The tree drawing (Fig. 1) and personality trait terms, described below, were used to measure participants’ attitudes toward elderly adults and people other than elderly adults.

#### Personality trait terms

These terms were used to evaluate the impression of the tree drawer’s personality by participants. We selected terms from a short form of the Japanese Big Five Scale of personality trait adjectives [25]. The scale was developed based on Big Five personality traits—conscientiousness, agreeableness, extraversion, neuroticism, and openness—and consisted of 29 personality trait adjectives that were evaluated on a scale from 1 (‘not applicable at all’) to 7 (‘very applicable’). The validity of the scale was confirmed by exploratory factor analysis, and the reliability was confirmed by Cronbach’s alpha [25].

The 5 personality trait terms used in this study were selected from the 29 personality trait terms according to 3 criteria: (1) one term was chosen for each of the 5 personality traits, (2) suitable terms were chosen to evaluate the impression of the drawer’s personality, and (3) factor loading was prioritized in the original study [25]. The 5 personality trait terms were selected as follows: negligence was selected from conscientiousness, short-tempered was selected from agreeableness, cheerful was selected from extraversion, timid was selected from neuroticism, and broad interests was selected from openness. These terms were evaluated on a scale from 1 (‘not applicable at all’) to 7 (‘very applicable’) with the same method used in the original scale [25].

### Participants

Participants were university students at a school of allied health sciences in Japan. To estimate the sample size needed for adequate power, we selected a similar previous study that researched empathy toward elderly adults among nursing students [8]. From the data of the study, we assumed that the mean difference was 9.3 and the SD was 9.8 among groups. Based on a probability of a type I error (α) of < 0.05, a probability of a type II error (β) of < 0.80, and up to 10% missing values, the total required sample size was estimated to be 120 by the Bonferroni adjustment method (30 in each of the four conditions/groups). Therefore, we conducted a survey of 154 allied health students. All participants were students majoring in physical therapy, occupational therapy, speech therapy, orthoptics and visual science, and health science; additionally, all were second- and third-year students. Sample size calculation was performed using the R programming language and environment (R version 3.3.2) [26].

### Statistical analysis

Descriptive statistics for age, sex, and FSA-J score were calculated for all participants. Based on the mean FSA-J score among all participants, participants were divided into high- and low-FSA-J groups. Differences in age, sex, and FSA-J score between the display conditions and the groups based on FSA-J score were examined using t-tests and chi-square tests.

To investigate differences in the evaluation of the drawer’s personality score by the display conditions and the groups based on FSA-J, a two-way analysis of variance (ANOVA) was conducted. The independent variables were the display conditions (drawer-age display condition) and the groups based on FSA-J (high and low FSA-J), and the dependent variables were the 5 personality trait terms. A post hoc analysis using the G*Power program for two-way ANOVA was performed to calculate statistical power [27]. All significance levels were 0.05, and analyses without a post hoc analysis were performed using the IBM SPSS 22 statistical package.

## Results

### Descriptive statistics for all participants, display conditions, and groups based on the FSA-J (Table 1)

One hundred twenty-three of 154 students were selected for analysis because they had no missing items in their completed questionnaires. The mean age of the participants was 19.56 years (SD = 0.82). There were more females than males (female = 76.42%, χ^2^ = 34.35, df = 1, *p* < 0.001). The mean FSA-J score was 29.80 (SD = 8.62).

**Table 1:** Demographics and mean scores of the participants

	All participants *n* = 123	Groups based on FSA-J		Display conditions	
		Low *n* = 64	High *n* = 59	Elderly display condition *n* = 61	Control condition *n* = 62
Age, mean ± SD	19.56 ± 0.82	19.48 ± 0.91	19.64 ± 0.72	19.48 ± 0.97	19.64 ± 0.65
Sex, n (%)					
Female	94 (76.42)	53 (43.1)	41 (33.3)	44 (35.77)	50 (40.65)
Male	29 (23.58)	11 (8.9)	18 (14.6)	17 (13.82)	12 (9.76)
FSA-J, mean ± SD	29.80 ± 8.62	23.27 ± 4.32	36.90 ± 6.14	29.89 ± 7.57	29.73 ± 9.61

Of all participants, 61 were in the elderly display condition, and 62 were in the control condition. There were no significant differences between the elderly display and the control condition for age (*t* = 1.100, df = 120, n.s.), sex (χ^2^ = 1.24, df = 1, n.s.), and FSA-J score (*t* = 0.10, df = 121, n.s.).

Based on the mean FSA-J score, we divided all participants into a low-FSA-J group and a high-FSA-J group. The low-FSA-J group that scored 29 or under included 64 participants, and the high-FSA-J group that scored 30 or more included 59 participants. There were no significant differences between the low-FSA-J and high-FSA-J groups in age (*t* = 1.04, df = 117.90, n.s.) or sex (χ^2^ = 3.02, df = 1, n.s.). The mean FSA-J score in the high-FSA-J group was higher than that in the low-FSA-J group (*t* = 14.32, df = 121, *p* < 0.001).

### Differences in the evaluation of the drawer’s personality score by the display conditions and the groups based on the FSA-J

According to the groups based on the FSA-J score (low FSA-J/high FSA-J) and the display conditions (elderly display/control), the following four groups were formed: the elderly display condition was applied to 29 participants in the low-FSA-J group and 32 participants in the high-FSA-J group, and the control condition was applied to 35 participants in the low-FSA-J group and 27 participants in the high-FSA-J group.

**Fig. 2:**
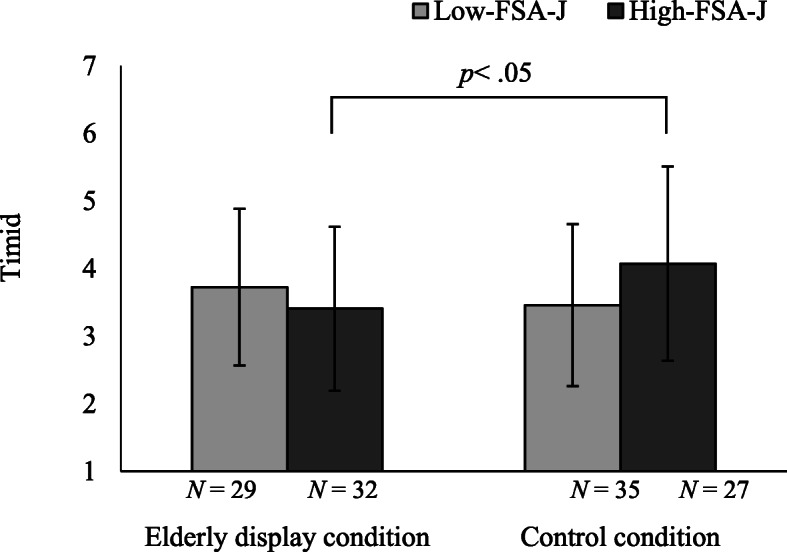
Means and standard deviations of timidity according to FSA-J and drawer–age display condition

Table 2 indicates the mean scores of the evaluation of the drawer’s personality based on 5 personality trait terms by these groups and conditions. In the high-FSA-J groups, the control condition evaluated the drawer’s personality as more timid than in the elderly display condition (*F* = 4.26, df = 1, 119, *p* < 0.05; see Fig. 2). Regarding negligence, the high-FSA-J group evaluated the drawer’s personality as more negligent than the low-FSA-J group (*F* = 4.08, df = 1, 119, *p* < 0.05). Regarding broad interests, participants in the elderly display condition evaluated the drawer’s personality as involving more broad interests than in the control condition (*F* = 4.23, df = 1, 119, *p* < 0.05). For all other items, there were no significant main effects or interactions. From the post hoc analysis for the ANOVA, 1-β was most likely 0.79.

**Table 2:** Mean scores and SDs of evaluations of the drawers’ personalities

	Elderly display condition		Control condition		Groups based on FSA-J		Display conditions		Result of ANOVA
	Low-FSA-J *n* = 29	High-FSA-J *n* = 32	Low-FSA-J *n* = 35	High-FSA-J *n* = 27	Low	High	Elderly display	Control	
Negligence	3.55 (1.18)	4.28 (1.28)	3.91 (1.52)	4.19 (1.44)	3.75 (1.38)	4.24 (1.34)	3.93 (1.28)	4.03 (1.48)	Main effect of FSA-J F = 4.08, df = 1, 119, *p* < 0.05
Short-tempered	4.41 (1.27)	4.25 (1.34)	4.26 (1.42)	4.44 (1.50)	4.33 (1.35)	4.34 (1.41)	4.33 (1.30)	4.34 (1.45)	n. s.
Cheerful	3.59 (1.43)	3.66 (1.31)	3.77 (1.42)	3.70 (1.30)	3.69 (1.41)	3.68 (1.29)	3.62 (1.36)	3.74 (1.35)	n. s.
Timid	3.72 (1.16)	3.41 (1.21)	3.46 (1.20)	4.07 (1.44)	3.58 (1.18)	3.71 (1.35)	3.56 (1.19)	3.73 (1.33)	Interaction *F* = 4.26, df = 1, 119, *p* < 0.05
Broad interests	4.00 (1.28)	3.75 (1.08)	3.20 (1.08)	3.70 (1.10)	3.56 (1.23)	3.73 (1.08)	3.87 (1.18)	3.42 (1.11)	Main effect of condition *F* = 4.23, df = 1, 119, *p* < 0.05

## Discussion

The present study examined ageism among allied health students, with the hypothesis that ageism was expressed not only toward elderly adults but also toward those other than elderly adults. The ageism score in the study was thought to be an average score among allied health students in Japan [24], the sample size requirements were satisfied, and 1-β was most likely adequate [28].

The results of this study suggested that ageism among allied health students contributed to their expression of negative evaluations, not only among elderly adults but also among those other than elderly adults. If ageism contributed to a negative evaluation of only the elderly, participants with high FSA-J in the elderly display condition evaluated negatively. However, there was no significant difference in personality evaluations between the low- and high-FSA-J groups in the elderly display condition. The result of negligence showed the possibility that ageism influenced people beyond the elderly adult population. Overall, participants with high levels of negative ageism provided negative evaluations regardless of the drawer’s age. It is possible that ageism among students might have roots in their personality. This suggestion was reported by a study that showed a correlation between the participants’ personality and their ageism [19]. When negative ageism influences interpersonal evaluations in general, ageism-related education is needed to consider allied health students’ personalities and evaluations, not only for elderly adults but also for those other than elderly adults.

Additionally, the following two results in this study suggested that participants with negative ageism were concerned with positive evaluations of the elderly. Regarding the quality timid, students with negative ageism evaluated the older drawer more positively than when the drawer’s age was concealed. Despite high negative ageism, students who knew that the drawer was elderly provided positive evaluations. For broad interests, students who knew that the drawer was elderly evaluated the drawer more broadly than participants who did not know the drawer’s age.

In other words, conscious ageism, which was measured by the FSA-J, revealed positive evaluations toward the elderly. This finding could have several explanations, such as the measurement of the methods for ageism and reaction formation. For example, it might be not enough to measure ageism using only the FSA-J. The FSA-J was designed to measure negative and conscious attitudes toward elderly adults [23]. However, ageism is complicated because there are several types, such as explicit and implicit attitudes [29] and negative and positive attitudes [30]. In particular, allied health students in Japan do not always have negative prejudices toward elderly adults [23, 24], although medical staff sometimes have negative attitudes toward elderly adults [31–35]. According to the results in this study and to previous suggestions, we have assumed that negative ageism among allied health students in Japan might underlie positive stereotypes. This finding led to the possibility that allied health students in Japan convince themselves that they have to have a positive attitude toward elderly adults. This hypothesis can also be considered a reaction formation.

In any case, these results might be caused by the methods in this study, which investigated attitudes not only toward elderly adults but also toward those other than elderly adults. Further study is required to consider the effect of positive stereotypes of elderly adults among allied health students.

### Limitations

The procedure in this study has some limitations. This study used a tree drawing to evaluate the target’s personality due to restricting the influence on stereotypical attitudes. A vignette experiment is a general method to evaluate social evaluation. The method used for tree drawing might be difficult to evaluate for the students. Second, this study used only 5 trait items for the evaluation. Accordingly, future studies must use the general method for evaluation and several items for multiple evaluation.

## Conclusions

The results of this study showed that allied health students with negative ageism provide negative evaluations not only toward elderly adults but also toward those other than elderly adults. It is possible that negative ageism has roots in personality. Therefore, education about ageism should consider students’ personalities and attitudes, not only toward elderly adults but also toward those other than elderly adults. Moreover, as allied health students in Japan with negative ageism do not always provide negative evaluations, it was thought that negative stereotypes and positive stereotypes are closely tied to each in this student population.

## Supplementary Information

**Additional file 1.** The questionnaire for the elderly display condition.

**Additional file 2.** The questionnaire for the control condition.

## Data Availability

The data is available from the openICPSR database (DOI: 10.3886/E121041V1).

## Abbreviations

FSA-J: Japanese short version of the Fraboni Scale of Ageism
ANOVA: Analysis of variance
