# Supplementary material for: The influence of ageism on stereotypical attitudes among allied health students in Japan: a group comparison design
Source: BMC Med Educ. 2021 Jan 7;21:27. doi: 10.1186/s12909-020-02439-0 (PMC7792127; doi:10.1186/s12909-020-02439-0)
Supplement: Supplementary file 2 — Additional file 2. The questionnaire for the control condition. [file 12909_2020_2439_MOESM2_ESM.pdf]

1. Please surmise the personality of the person who drew this tree.

	Not applicable at all	Not applicable	Not very applicable	Not sure	Somewhat applicable	Applicable	Very applicable
(1) NEGLIGENCE	1	2	3	4	5	6	7
(2) SHORT-TEMPERED	1	2	3	4	5	6	7
(3) CHEERFUL	1	2	3	4	5	6	7
(4) TIMID	1	2	3	4	5	6	7
(5) BROAD INTERESTS	1	2	3	4	5	6	7

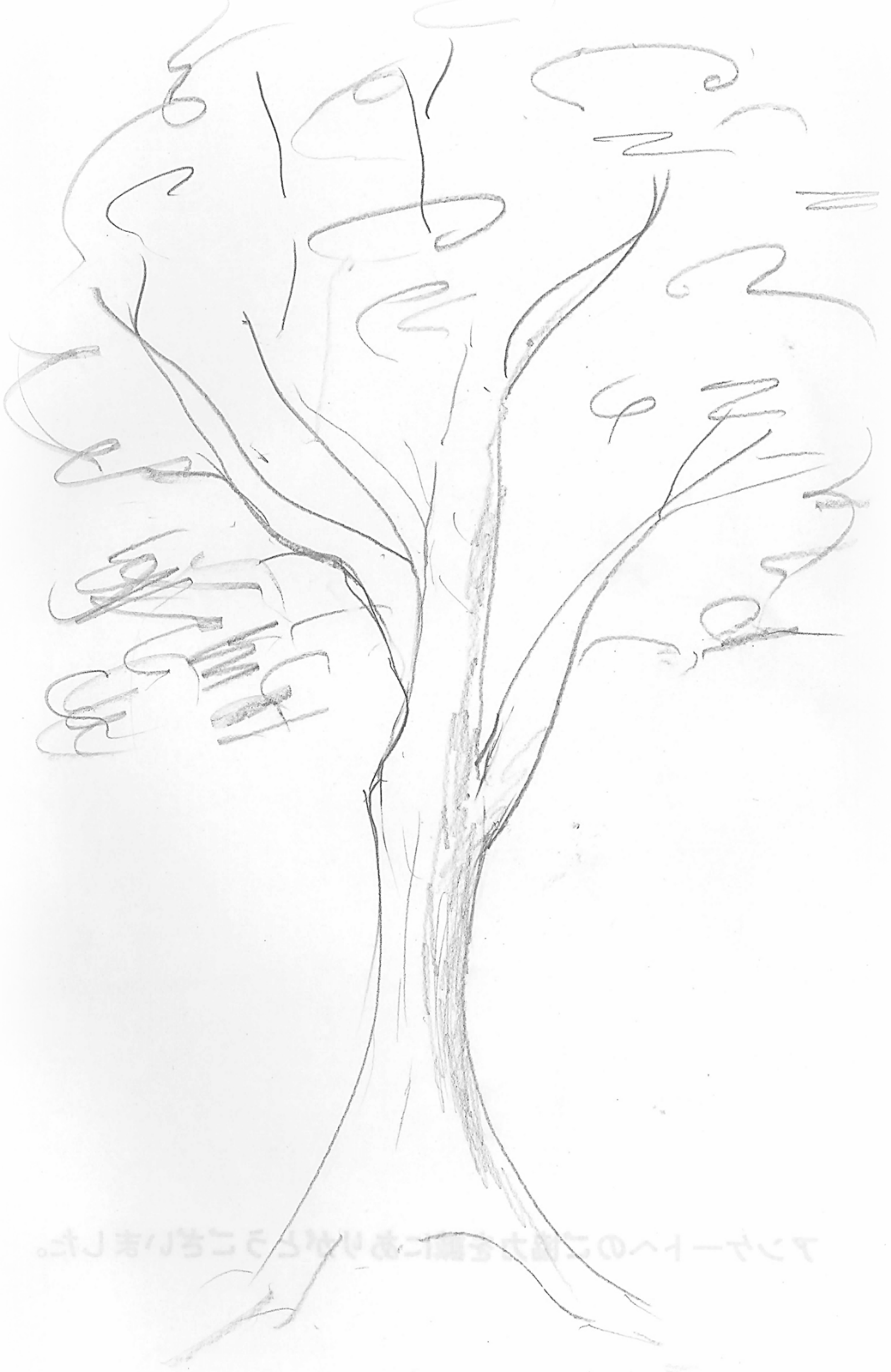

2. Let me know your view about 65 years of age or over.

Next to each item, please the number that best describes your answer.

	Strongly disagree	Disagree	Neither agree nor disagree	Agree	Strongly agree
(1) Many old people are stingy and hoard their money and possessions.	1	2	3	4	5
(2) Many old people are not interested in making new friends preferring instead the circle of friends they have had for years.	1	2	3	4	5
(3) Many old people just live in the past.	1	2	3	4	5
(4) I sometimes avoid eye contact with old people when I see them.	1	2	3	4	5
(5) I don't like it when old people try to make conversation with me.	1	2	3	4	5
(6) Old people should feel welcome at the social gatherings of young people.	1	2	3	4	5
(7) I would prefer not to go to an open house at a senior's club, if invited.	1	2	3	4	5
(8) I personally would not want to spend much time with an old person.	1	2	3	4	5
(9) Most old people should not be allowed to renew their driver's licenses.	1	2	3	4	5
(10) Old people don't really need to use our community sports facilities	1	2	3	4	5
(11) Most old people should not be trusted to take care of infants.	1	2	3	4	5
(12) It is best that old people live where they won't bother anyone.	1	2	3	4	5
(13) The company of most old people is quite enjoyable.	1	2	3	4	5
(14) It is sad to hear about the plight of the old in Japanese society these days.	1	2	3	4	5
(15) Old people should be encouraged to speak out politically.	1	2	3	4	5
(16) Most old people are interesting, individualistic people.	1	2	3	4	5
(17) I would prefer not to live with an old person.	1	2	3	4	5
(18) Most old people can be intimidating because they tell the same stories over and over.	1	2	3	4	5
(19) Old people complain more than other people do.	1	2	3	4	5

3. Please fill or check in your information below.

Age………(　　　　)

Grade……… (　　　　)

Sex……… (Male ・ Female)
